# Optical Coherence Tomography Angiography Findings Associated With Accelerated Hypertension

**DOI:** 10.7759/cureus.59290

**Published:** 2024-04-29

**Authors:** Salil Mehta

**Affiliations:** 1 Ophthalmology, Lilavati Hospital, Mumbai, IND

**Keywords:** fundoscopy, hypertension, ocular, optical coherence tomography angiography, accelerated hypertension

## Abstract

Introduction

Accelerated hypertension, that is a systolic blood pressure greater than 180 mmHg and a diastolic blood pressure greater than 120 mmHg is often accompanied by fundoscopic signs with the potential of systemic and visual morbidity. We report on the clinical and optical coherence tomography angiography (OCTA) findings in a cohort of hypertensive patients with accelerated hypertension.

Methods

Patients, presenting to the emergency room/intensive care unit, who met the clinical definition of accelerated hypertension (a blood pressure >180/120 mmHg,), were triaged to the intensive care unit. Following blood pressure reduction via pharmacological methods, a standard panel of hematological tests, cardiac evaluation tests, and the necessary systemic imaging was performed. They underwent a bedside dilated fundus examination with subsequent fundus photography/OCTA using a Topcon DRI OCT plus (Topcon Corporation, Tokyo, Japan). The records of these patients were evaluated.

Results

We analyzed the records of 16 patients (12 males (75%), and four females (25%)) with ages ranging from 16 to 75 years (mean 47.6 years). Eleven patients consented to a detailed evaluation. These included nine males (81.8%) and two females (18.1%) with ages ranging from 16 to 63 years (mean 46.3 years). Comorbidities included pre-existing hypertension (nine patients, 81.8%), chronic kidney disease (three patients, 27.2%), and diabetes mellitus type 2 (two patients, 18.1%). Clinical findings in these 22 eyes included arteriolar changes consistent with Keith Wagener Barker (KWB) grade 1 (two eyes, 9.0%), grade 2 (10 eyes, 45.4%), grade 3 (eight eyes, 36.3%), and grade 4 (two eyes, 9.0%). OCTA findings included capillary nonperfusion in the superficial capillary plexus in the areas of retinal opacification (seven eyes, 31.8%).

Conclusion

OCTA studies of the macular, as well as the entire posterior pole vasculature, may help to detect retinal microangiopathy, permit accurate grading, and subsequently develop a model that permits the quantification of systemic and ocular risk in these patients.

## Introduction

Accelerated hypertension or malignant hypertension is defined as the recent onset of a significant increase in blood pressure that may lead to end-organ injury. While definitions vary, a systolic blood pressure greater than 180 mmHg and a diastolic blood pressure greater than 120 mmHg are currently accepted as significant [1]. This elevation is often accompanied by fundoscopic evidence of angiopathic damage such as arteriolar narrowing, hemorrhages, cotton wool spots, or papilledema with the potential for visual morbidity [2]. An optical coherence tomography angiography (OCTA) study allows for the rapid and non-invasive evaluation of these findings especially the capillary plexus and retinal blood flow and several studies have reported on the OCTA findings in patients with patients with chronic systemic hypertension but not specifically accelerated hypertension. We report on the clinical and OCTA findings in a cohort of hypertensive patients with accelerated hypertension (blood pressure on admission greater than 180/120 mmHg).

## Materials and methods

This is an IRB-approved retrospective observational study. Patients, presenting to the emergency room/intensive care unit who met the clinical definition of accelerated hypertension (a blood pressure >180/120 mmHg), were triaged to the intensive care unit. Following a detailed clinical evaluation by a cardiologist/intensivist, prompt measures were undertaken to reduce the blood pressure usually via the use to intravenous nitroglycerin (NTG) in an appropriate dose. A comprehensive evaluation was carried out that consisted of a complete blood count, renal function tests, liver function tests, troponin T and I, creatine phosphokinase-MB (CPK-MB), N-terminal pro-B-type natriuretic peptide (NT-Pro BNP), urinary vanillylmandelic acid (VMA) (24 hours), and serum catecholamines. Additionally, they underwent 2D echocardiography, electrocardiogram, and renal artery Doppler. Patients underwent a dilated fundus examination at the bedside usually the next day, using an indirect ophthalmoscope. Patients with detectable retinopathy (of any grade) were advised a more detailed evaluation. Those who consented underwent visual acuity testing (Snellen’s chart), slit lamp evaluation of the anterior segment, and an OCT/OCTA study, specifically, 9x9 mm and/or 12x12 mm scans to evaluate the entire posterior pole. We used a Topcon DRI OCT plus (Topcon Corporation, Tokyo, Japan) for this evaluation. This is a swept-source OCT that uses a 1050 nm wavelength to generate images.

We used Keith, Wagner, and Barker’s (KWB) classification which classified changes into grade 1 (mild narrowing or sclerosis of retinal arterioles); grade 2 (moderate to severe retinal arteriolar narrowing with venous compressions at A-V crossing); grade 3 marked by retinal hemorrhages, exudates and cotton wool spots and grade 4 marked by papilledema, for the clinical grading of any detected hypertensive retinopathy [3].

## Results

We analyzed the records of 16 patients (12 males (75%), and four females (25%)) with ages ranging from 16 to 75 years (mean 47.6 years). Of these patients, 12 (75%) were hypertensive, five (41.6%) were diabetic (type 2), and three (18.7%) had pre-existing chronic kidney disease. Clinical findings in these 32 eyes included arteriolar changes consistent with KWB grade 1 (eight eyes, 25%), grade 2 (14 eyes, 43.7%), grade 3 (eight eyes, 25%), and grade 4 (two eyes, 6.2%). Additional findings included non-proliferative diabetic retinopathy (four eyes, 12.5%) and stable retinopathy post-panretinal photocoagulation (two eyes, 6.2%). Three patients (18.7%) had systemic end-organ damage consistent with accelerated hypertension, one patient (case 7, 33%) with an intracerebral hemorrhage, and two patients with chronic kidney disease (cases 2, 3 (66%)).

Eleven patients consented to a detailed evaluation. These included nine males (81.8%) and two females (18.1%) with ages ranging from 16 to 63 years (mean 46.3 years). Comorbidities included pre-existing hypertension (nine patients, 81.8%), chronic kidney disease (three patients, 27.2%), and diabetes mellitus type 2 (two patients, 18.1%). Clinical findings in these 22 eyes included arteriolar changes consistent with KWB grade 1 (two eyes, 9.0%), grade 2 (10 eyes, 45.4%), grade 3 (eight eyes, 36.3%) and grade 4 (two eyes, 9.0%). OCTA findings included capillary nonperfusion in the superficial capillary plexus in the areas of retinal opacification (seven eyes, 31.8%). One patient (case 10) was initially diagnosed as grade 4 (papilledema) but the absence of hemorrhages/exudates prompted a search for an alternative diagnosis. A subsequent lumbar puncture revealed an opening pressure of 40 cmH_2_O (normal 7-18 cmH_2_O) with normal cerebrospinal fluid (CSF) examination findings confirming a diagnosis of idiopathic intracranial hypertension.

These findings are summarized in Table 1.

**Table 1:** Significant findings of the clinical and OCTA findings of the cohort. KWB: Keith- Wagner-Barker; HTN: hypertension; CKD: chronic kidney disease; SP: superficial plexus; OCTA: optical coherence tomography angiography

No	Age	Sex	Comorbidities	Fundus R	Fundus L	OCT/OCTA R	OCT/OCTA L
1	63	M	HTN	KWB 2	Retinal opacification/hemorrhages (KWB 3)	n	capillary dropout in SP
2	16	M	CKD St 5	Multiple hemorrhages/exudates (KWB 3)	Multiple hemorrhages/exudates (KWB 3)	Capillary nonperfusion in SP	Capillary nonperfusion in SP
3	46	M	HTN/acute coronary syndrome/CKD	Retinal opacification (KWB 3)	Retinal opacification (KWB 3)	Capillary nonperfusion in SP	Capillary nonperfusion in SP
4	42	M	HTN/AKI/no DM	KWB 4/papilledema	KWB 4/papilledema	n	n
5	63	M	HTN/borderline DM	KWB1	KWB1	n	n
6	31	M	HTN/borderline dm	KWB 3	KWB 3	Peripapillary edema	peripapillary edema
7	56	F	HTN/lentiform nucleus hematoma	KWB 2	KWB 2	Capillary nonperfusion in SP	n
8	43	M	HTN	KWB 2	KWB 2	n	n
9	46	F	HTN	KWB 2	KWB 2	n	n
10	57	M	HTN/CKD	KWB 2 +(papilledema)	KWB 2 + (papilledema)	n	n
11	30	M	Nil	KWB 2	KWB 3 (retinal opacification)	n	Capillary nonperfusion in SP

We report the clinical and ocular findings of two patients who were seen in the emergency room and were subsequently diagnosed with accelerated hypertension. Their characteristic fundus and ocular imaging findings permitted an accurate grading of the retinopathy and assisted the cardiologist in therapeutic planning.

Case report one

**Figure 1:**
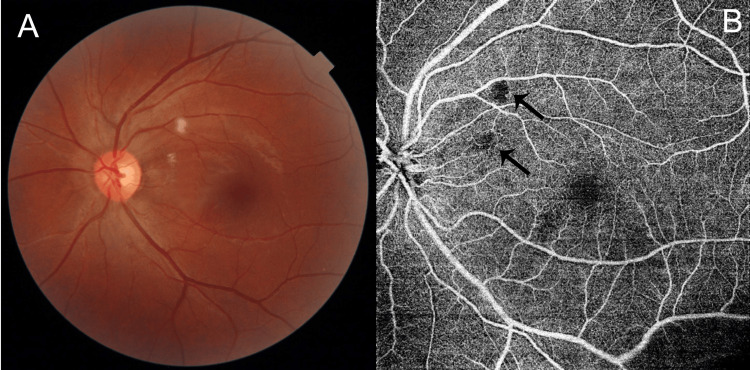
Case 11 images A: Fundus photo showing areas of retinal opacification in the posterior pole of the left eye. B: OCTA image showing areas of capillary dropout within the superficial capillary plexus (black arrows). OCTA: optical coherence tomography angiography

A 30-year-old male patient, not a previously known hypertensive or diabetic, presented with persistent dry cough in the past two to three weeks and chest discomfort for one week. This was accompanied by dyspnea which increased on exertion. He underwent a routine chest X-ray which revealed cardiomegaly and was transferred to our center for further management. On admission, he was conscious and oriented. He was febrile, with a pulse rate of 99/min and normal findings from an examination of the nervous, respiratory, and cardiovascular systems. His blood pressure was 190/148 mmHg and he was started on an intravenous infusion of NTG (50 mg in 50 cc normal saline at 1 ml/h) till control was achieved. A clinical diagnosis of acute coronary syndrome secondary to hypertension was made. Relevant investigations included a normal complete blood count (hemoglobin estimation 12.3 g/dl (normal 13.0-17.0 g/dl), white blood cell count 9800/mm^3^ (normal 4000-10000/µl); platelet count 150,000/mm^3^ (normal 150,000-410,000 /µl) but altered renal functions (blood urea nitrogen 23.30 mg/dl (normal 7-20 mg/dL) and serum creatinine 1.83 (normal 0.7-1.4 mg/dl)). The estimation of N-terminal proBNP (NT-ProBNP) was 11,486 pg/ml (normal < 125 pg/ml). A 2D echo revealed left ventricular and atrial hypertrophy with generalized left ventricular hypokinesia suggestive of a “stunned myocardium” with an ejection fraction of 10-15%. Ophthalmic examination showed a normal visual acuity bilaterally and normal slit lamp examination bilaterally. Dilated fundus examination was normal in the right eye and revealed multiple areas of retinal opacification in the posterior pole of the left eye. An OCT scan through these areas revealed hyperreflectivity in the inner retinal layers with areas of capillary nonperfusion in the superficial capillary plexus on OCTA corresponding to the areas of opacification (Figure 1). He was subsequently discharged on a regimen of oral metoprolol (25 mg thrice daily); oral cilnidipine (10 mg twice a day), and oral prazosin (5 mg twice a day).

Case report two

**Figure 2:**
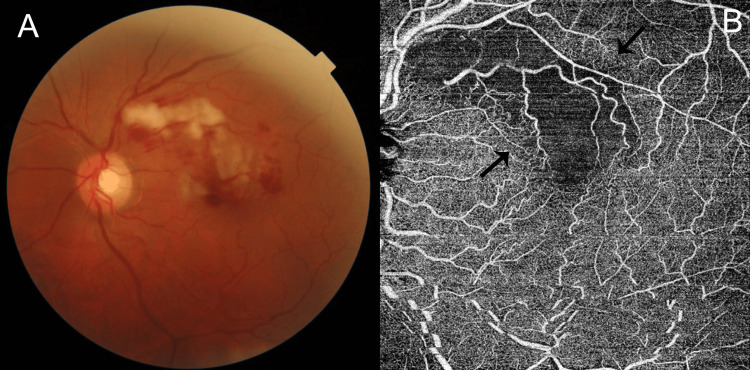
Case 1 images A: Fundus photo showing areas of extensive retinal opacification in the posterior pole of the left eye. B: OCTA image showing areas of capillary dropout within the superficial capillary plexus (black arrows). OCTA: optical coherence tomography angiography

A 63-year-old non-diabetic male, a previously known hypertensive, with voluntary cessation of his treatment for the last three years, presented to our emergency with feelings of uneasiness for one day. On evaluation, he was conscious and oriented. He was afebrile, with a pulse rate of 94/min and normal findings from an evaluation of the nervous, respiratory, and cardiovascular systems. His blood pressure was 190/90 mmHg and he was started on an intravenous infusion of NTG (50 mg in 50 cc normal saline at 1 ml/h) till normotensive control was achieved. Relevant investigations included a normal complete blood count (hemoglobin estimation (14.7 g/dl; normal 13.0-17.0 g/dl); WBC 6600/mm^3^ (normal 4000-10000/µl); platelet count 206,000/mm^3^ (normal 150,000-410,000/µl) and normal renal functions (blood urea nitrogen 14.40 mg/dl (7-20 mg/dl) and serum creatinine 1.13 mg/dl (normal 0.7-1.4 mg/dl)). The estimation of NT-ProBNP was normal at 70.60 pg/ml (normal < 125 pg/ml). 2D echo revealed no regional wall motion abnormalities with an ejection fraction of 60%. An ECG was normal. Ophthalmic examination showed a normal visual acuity (6/6, N6) in the right eye and 6/12, N8 in the left eye. Slit lamp examination was normal bilaterally. Dilated fundus examination was normal in the right eye and revealed multiple areas of retinal opacification with extensive superficial retinal hemorrhages in the posterior pole of the left eye. An OCT scan through these areas revealed hyperreflectivity in the inner retinal layers with areas of capillary nonperfusion in the superficial capillary plexus on OCTA corresponding to the areas of opacification and hemorrhage (Figure 2). He was subsequently discharged on a regimen of oral antihypertensives (bisoprolol 5 mg once a day; amlodipine 5 mg once a day).

## Discussion

Several definitions of acute severe hypertension exist. Some authors suggest using the term “acute severe hypertension” when there is a severe elevation in blood pressure but without signs of life-threatening end-organ damage. A hypertensive emergency is an elevated blood pressure that could potentially lead to conditions such as pulmonary edema or acute kidney injury or intracerebral bleeding. Malignant hypertension is a type of severe hypertension (usually > 200/120 mmHg) marked by vascular damage as result of auto-regulatory failure and a clinical diagnosis mandates the detection of grade 3 or grade 4 hypertensive retinopathy [1]. We used a broad definition (>180/120 mmHg) to identify patients with accelerated hypertension with or without evidence of end-organ injury.

Accelerated hypertension occurs when there is a rapid rise in blood pressure in a short span of time. There is an increase in systemic vascular resistance which occurs via the renin-angiotensin pathway or hypoperfusion. Within the retina, the early effects of a rise in blood pressure result in vasospasm to autoregulate perfusion [2]. This has its clinical correlates in arteriolar narrowing with the appearance of retinal opacification/hemorrhages in the group of patients with accelerated hypertension. These have their imaging correlates in the areas of capillary nonperfusion we observed.

Several large studies have described the fundus and imaging findings in patients with chronic hypertension. Lee et al. analyzed 58 healthy controls, 37 patients with chronic hypertension without retinopathy, and 31 patients with relieved grade 4 hypertensive retinopathy. The central macula, retinal nerve fiber layer (RNFL), and ganglion cell-inner plexiform layer (GCIPL) in both the latter groups were thinner than the controls [4].

Hua and co-workers studied 73 and 40 healthy volunteers in four groups: Group A (patients with intensive BP control); Group B (patients with standard BP control); Group C (patients with poor BP control); and Group D (control group). They studied the results of 6 × 6 mm macula scans and 4.5 × 4.5 mm optic nerve head scans and concluded that retinal vascular density was significantly reduced in patients with poor BP control as well as standard BP control as compared to patients on intensive BP control and the control group [5].

Liu et al. analyzed a 3 × 3-mm macular map of 100 patients with hypertensive retinopathy and 66 volunteers. They described five predominant findings which included focal capillary sparsity, scattered microangioma, focal macular arch ring defects, focal capillary disorder, and focal capillary nonperfusion [6].

A limited number of case reports have described the OCTA findings of patients with acute severe hypertension. Mirshahi and co-workers reported the case of a 41-year-old male with bilateral visual loss after an elevation of his blood pressure (200/150 mmHg). Fundus examination revealed disk edema, arteriolar narrowing, hemorrhages, and exudates. An OCTA scan revealed disruption in the normal vascularity of the superficial and deep capillary plexuses whereas a fluorescein angiogram revealed significantly less vascular detail [7].

Rotsos et al. described the OCT/OCTA (3x3mm, 6x6 mm scans) findings of two patients with acute hypertension. Case one was a 46-year-old woman with hemorrhages and Elschnig spots. An OCT scan revealed serous retinal detachment and hyperreflective structures anterior to the retinal pigment epithelium. These structures were noted as hyperreflective lesions on OCTA. Case two described a 35-year-old woman with optic disk edema, hemorrhages, and Elschnig spots. An OCT scan revealed subretinal fluid and solid hyperreflective structures. OCTA scans did not reveal any abnormal areas in the choriocapillaris layer [8].

Published literature describing the OCTA findings in hypertensive choroidopathy is less common. Karagiannis et al. described the case of a 51-year-old hypertensive (> 200 mmHg systolic) female patient who presented with reduced visual acuity in the left eye, OCT findings of an exudative retinal detachment and OCTA findings demonstrating a flow void in the choriocapillaris suggestive of choroidal ischemia. All these findings returned to a normal state at two monthly follow-up following stabilization of her blood pressure [9].

Rezkallah and co-workers described the case of a 25-year-old white woman with renal failure and intracranial hypertension who presented with bilateral visual loss. The fundus examination revealed bilateral arterial narrowing and venous dilation. Additionally, deep yellow spots were seen along with exudative retinal detachments. An OCTA analysis was done which revealed extensive flow voids in the choriocapillaris slabs bilaterally. A follow-up at one month revealed a significant reduction in the number of these voids. The authors suggest that that the acute elevation of blood pressure leads to choroidal ischemia which has imaging correlates in the choriocapillaris flow voids. As the acute elevation of blood pressure and the concurrent choroidal ischemia are ameliorated, these voids are then seen to reduce [10].

Similar findings were described by Viruni et al. who reported on the clinical and multimodal imaging findings of an 87-year-old woman with uncontrolled hypertension. A fundus examination in her left eye revealed vascular tortuosity with hemorrhages and Elschnig spots. An OCTA scan revealed extensive flow voids in the choriocapillaris layer as well as capillary nonperfusion areas in the retinal plexus. At a three-monthly follow-up, her visual acuity improved with a reduction in the flow voids of the choriocapillaris but the capillary nonperfusion of the retinal plexus was unchanged. The authors suggest that hypertension-induced fibrinoid necrosis of choroidal arterioles with a breakdown of the blood-retinal barrier may account for these changes [11].

These patients are susceptible to coronary artery disease, peripheral vascular disease, and cerebrovascular events due to similar damage in other end-organ systems. Ong et al used retinal photographs within the Atherosclerosis Risk in Communities (ARIC) Study which studied 15,792 participants and noted that hypertensive retinopathy was associated with an increased risk of stroke, independent of other risk factors. They recommended the use of fundus examination to help assess the risk of stroke in these patients. [12]. Chen and co-workers studied 9793 hypertensive patients within the China Stroke Primary Prevention Trial. Of these, 592 patients, suffered from a stroke at the end of a 4.4-year follow-up, and a significant association was noted between the presence of hypertensive retinopathy and the risk of a cerebrovascular event [13].

Hua and co-workers noted that the microvascular structure of the retina shares numerous similarities with cerebral and cardiac tissue and fundus examination may have an important role in the detection of end-organ damage. They suggest that the use of OCTA could increase the prediction of end-organ damage in target organs [14].

Additionally, the persistence of arteriolar sclerosis can potentially lead to an increased incidence of arterial or vein occlusions within the eye [15].

We report the clinical and OCTA findings of 11 patients with accelerated hypertension to assess the retinal vascular damage as a result of the acute rise in blood pressure. We opted for 9x9 mm and 12x12 mm scans to permit analysis of the entire posterior pole where the majority of lesions were located as compared to a limited macular vascular pattern analysis. The commonest finding was capillary nonperfusion of the superficial capillary plexus corresponding to the areas of visible retinal opacification. We could detect no abnormalities in the deep capillary plexus, choriocapillaris, or choroidal layers. One patient had peripapillary edema within the outer nuclear layer but a normal OCTA.

Our findings were similar to the patients described by Mirshahi et al. [7] who described similar patterns of capillary dropout within the superficial and deep retinal plexus, and Viruni et al. [11] who described a similar capillary dropout within the retinal capillary plexus in association with choriocapillaris flow voids. Unlike the cases described by Rotsos and co-workers [8], we could not detect any hyperreflective lesions within the choriocapillaris.

Our study depended on the referral of patients from the cardiology/emergency room physicians and a referral bias toward more severe cases is possible. We did not assess macular vascular patterns and we agree that this remains an area of study in the future, especially in patients with long-term follow-up. The role of OCTA includes identifying areas of vascular pathology (especially nonperfusion), evaluation of individual plexuses and macular vascular patterns, and assessment of the perifoveal endocapillary area and its correlation to systemic blood pressure. This would help develop imaging biomarkers to quantify potential ocular and systemic morbidity and mortality risks within an Indian population.

## Conclusions

We report the clinical and OCTA findings in this cohort of 11 patients, some of whom had additional systemic end-organ damage. Clinical findings include hypertensive retinopathy ranging from KWB grade 1 to grade 4 and OCTA imaging findings that reveal superficial capillary plexus ischemia that correlates to the clinically visible lesions. OCTA imaging offers a non-invasive, reproducible method of vascular structure and function assessment within the retina. Further studies of the macular as well as the entire posterior pole vasculature may help to detect retinal microangiopathy, permit accurate grading, and subsequently develop a model that permits the quantification of systemic and ocular risk in these patients.

## References

[1] Kulkarni S, Glover M, Kapil V (2023). Management of hypertensive crisis: British and Irish Hypertension Society Position document. J Hum Hypertens.

[2] Di Marco E, Aiello F, Lombardo M (2022). A literature review of hypertensive retinopathy: Systemic correlations and new technologies. Eur Rev Med Pharmacol Sci.

[3] Tsukikawa M, Stacey AW (2020). A review of hypertensive retinopathy and chorioretinopathy. Clin Optom (Auckl).

[4] Lee SH, Lee WH, Lim HB, Jo YJ, Kim JY (2019). Thicknesses of central macular, retinal nerve fiber, and ganglion cell inner plexiform layers in patients with hypertension. Retina.

[5] Hua D, Xu Y, Zhang X, He T, Chen C, Chen Z, Xing Y (2021). Retinal microvascular changes in hypertensive patients with different levels of blood pressure control and without hypertensive retinopathy. Curr Eye Res.

[6] Liu Y, Li J, Pan J, Wang Y, Mao G, Jiang Z (2021). Morphological changes in and quantitative analysis of macular retinal microvasculature by optical coherence tomography angiography in hypertensive retinopathy. Hypertens Res.

[7] Mirshahi A, Karkhaneh R, Roohipour R, Rajabi M, Vahedian Z, Bazvand F (2022). Optical coherence tomography angiography findings in malignant hypertensive retinopathy. J Ophthalmic Vis Res.

[8] Rotsos T, Andreanos K, Blounas S, Brouzas D, Ladas DS, Ladas ID (2017). Multimodal imaging of hypertensive chorioretinopathy by swept-source optical coherence tomography and optical coherence tomography angiography: Case report. Medicine.

[9] Karagiannis D, Bouratzis N, Kontomichos L, Pantazis P, Kandarakis S, Paroikakis E (2023). Unilateral hypertensive choroidopathy as a sole manifestation in malignant hypertension: Optical coherence tomography angiography findings-case report. BMC Ophthalmol.

[10] Rezkallah A, Kodjikian L, Abukhashabah A, Denis P, Mathis T (2019). Hypertensive choroidopathy: Multimodal imaging and the contribution of wide-field swept-source oct-angiography. Am J Ophthalmol Case Rep.

[11] Viruni N, Ong SS, Wu JH, Liu TY (2022). Longitudinal optical coherence tomography angiography findings in malignant hypertension choroidopathy: A case report. Case Rep Ophthalmol.

[12] Ong YT, Wong TY, Klein R (2013). Hypertensive retinopathy and risk of stroke. Hypertension.

[13] Chen X, Liu L, Liu M (2021). Hypertensive retinopathy and the risk of stroke among hypertensive adults in China. Invest Ophthalmol Vis Sci.

[14] Hua D, Xu Y, Zeng X (2020). Use of optical coherence tomography angiography for assessment of microvascular changes in the macula and optic nerve head in hypertensive patients without hypertensive retinopathy. Microvasc Res.

[15] Kotruchin P, Tangpaisarn T, Mitsungnern T (2022). Hypertensive emergencies in Asia: A brief review. J Clin Hypertens (Greenwich).

